# Human umbilical cord mesenchymal stem cell‐derived exosomes attenuate neuroinflammation and oxidative stress through the NRF2/NF‐κB/NLRP3 pathway

**DOI:** 10.1111/cns.14454

**Published:** 2023-09-12

**Authors:** Ji Che, Hui Wang, Jing Dong, Yuanyuan Wu, Haichao Zhang, Lei Fu, Jun Zhang

**Affiliations:** ^1^ Department of Anesthesiology Fudan University Shanghai Cancer Center Shanghai China; ^2^ Department of Oncology, Shanghai Medical College Fudan University Shanghai China; ^3^ Shanghai Key Laboratory of Clinical Geriatric Medicine, Huadong Hospital Fudan University Shanghai China

**Keywords:** human umbilical cord mesenchymal stem cell (hUC‐MSC)‐derived exosomes, microglia, neuroinflammation, NLRP3, oxidative stress

## Abstract

**Aims:**

We investigated whether human umbilical cord mesenchymal stem cell (hUC‐MSC)‐derived exosomes bear therapeutic potential against lipopolysaccharide (LPS)‐induced neuroinflammation.

**Methods:**

Exosomes were isolated from hUC‐MSC supernatant by ultra‐high‐speed centrifugation and characterized by transmission electron microscopy and western blotting. Inflammatory responses were induced by LPS in BV‐2 cells, primary microglial cultures, and C57BL/6J mice. H_2_O_2_ was also used to induce inflammation and oxidative stress in BV‐2 cells. The effects of hUC‐MSC‐derived exosomes on inflammatory cytokine expression, oxidative stress, and microglia polarization were studied by immunofluorescence and western blotting.

**Results:**

Treatment with hUC‐MSC‐derived exosomes significantly decreased the LPS‐ or H_2_O_2_‐induced oxidative stress and expression of pro‐inflammatory cytokines (IL‐6 and TNF‐α) in vitro, while promoting an anti‐inflammatory (classical M2) phenotype in an LPS‐treated mouse model. Mechanistically, the exosomes increased the NRF2 levels and inhibited the LPS‐induced NF‐κB p65 phosphorylation and NLRP3 inflammasome activation. In contrast, the reactive oxygen species scavenger NAC and NF‐κB inhibitor BAY 11–7082 also inhibited the LPS‐induced NLRP3 inflammasome activation and switched to the classical M2 phenotype. Treatment with the NRF2 inhibitor ML385 abolished the anti‐inflammatory and anti‐oxidative effects of the exosomes.

**Conclusion:**

hUC‐MSC‐derived exosomes ameliorated LPS/H_2_O_2_‐induced neuroinflammation and oxidative stress by inhibiting the microglial NRF2/NF‐κB/NLRP3 signaling pathway.

## Graphical abstract

Human umbilical cord mesenchymal stem cell (hUC‐MSC)‐derived exosomes reduce neuroinflammation and oxidative stress in in vitro and in vivo models.

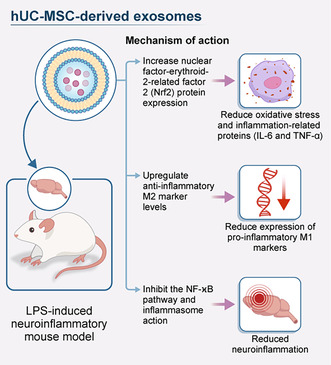

## 1 INTRODUCTION

Microglia activation resulting from the immune response produces excessive pro‐inflammatory cytokines and leads to oxidative stress, thus playing a pivotal role in various brain disorders.^1^ Lipopolysaccharide (LPS)‐stimulated microglial cells activate an inflammatory cascade, which releases inflammatory mediators that induce neuronal death, leading to sepsis‐associated encephalopathy (SAE).^2^ Moreover, these cells acquire pro‐inflammatory (classical M1) or anti‐inflammatory (classical M2) phenotypes upon activation. Pro‐inflammatory microglia releases inflammatory cytokines, such as nitric oxide (NO), interleukin‐1β (IL‐1β), and inducible nitric oxidase synthase (iNOS), whereas anti‐inflammatory microglial cells produce CD206, ARG1, YM1, and other protective molecules to promote neural tissue repair.^3^, ^4^ Neuroinflammation is common in multiple acute and chronic brain diseases, including stroke, Alzheimer's, and Parkinson's diseases.^5^ Therefore, novel therapeutic strategies are required to alleviate neuroinflammation and improve neurological outcomes.

Stem cell therapy is considered a promising approach owing to its regenerative and immunomodulatory ability. Human umbilical cord mesenchymal stem cells (hUC‐MSCs) have been widely used to treat numerous diseases,^6^ including chronic liver failure, nasopharyngeal carcinoma, arthritis, and COVID‐19. Exosomes are small extracellular vesicles of 30–150‐nm diameter^7^ that are present in the blood, urine, sperm, cerebrospinal fluid, breast milk, and almost all other body fluids.^8^ They contain bioactive molecules, such as proteins, lipids, microRNAs, and mRNAs,^9^ which are secreted by one cell type and endocytosed by another. Exosome‐mediated therapy is safer and more effective than stem cell therapy for certain diseases,^10^ such as stroke. Preclinical studies have demonstrated the exosome therapeutic effects in animal disease models,^11^ thereby highlighting their potential as clinical therapies.

Dental pulp stem cell‐ or bone marrow mesenchymal stromal cell‐derived exosomes could protect against ischemia–reperfusion‐induced brain damage.^12^ Further, hUC‐MSC‐derived exosomes have been used to protect rat retinal neurons under hyperglycemic conditions.^13^ Exosomes from different sources have exhibited neuroprotective potential against detrimental insults. These findings drove us to investigate whether exosomes have beneficial effects on neuroinflammation remission in the brain.

Inflammasomes are inducible high‐molecular‐weight protein complexes, involved in many pathological processes.^14^ The microglia NOD‐like receptor pyrin domain containing 3 (NLRP3) inflammasome is one of the most extensively studied inflammasomes in multiple neurodegenerative diseases. Activation of the NLRP3 inflammasome is emerging as a critical contributor to neuroinflammation and may be a potential target to treat neuroinflammation‐associated diseases. The NLRP3 inflammasome, consisting of the NLRP3 Apoptosis‐associated speck‐like (ASC) scaffold protein and Caspase‐1,^15^ can be activated by damage‐associated molecules consisting of adenosine triphosphate, high mobility group box‐1 (HMG1), monosodium urate, and reactive oxygen species (ROS)^16^ or pathogen‐derived LPS and lipoteichoic acid.^17^ Nuclear factor κB (NF‐κB), a key activator of inflammation, primes the NLRP3 inflammasome for activation by inducing *pro‐IL‐1β* and *NLRP3* expression. Therefore, activation of the NLRP3 inflammasome could be regulated by the NF‐κB signaling pathway.^15^ The NLRP3 inflammasome assembly causes the maturation and secretion of downstream inflammatory cytokines, such as IL‐1β and IL‐18,^15^, ^18^ which induces the inflammatory process. The Nuclear factor‐erythroid‐related factor 2 (NRF2) is an emerging drug target for neurodegenerative diseases.^19^ NRF2 nuclear translocation competes with NF‐κB p65 for the common transcriptional coactivator P300, thereby suppressing NF‐κB‐driven pro‐inflammatory responses.^20^ However, the contribution of NRF2 activation to hUC‐MSC‐derived exosome function in neuroinflammation remains elusive.

Therefore, in this study, we aimed to investigate whether exosomes derived from hUC‐MSCs could exert a neuroprotective effect on LPS‐induced neuroinflammation in vitro and in vivo. Furthermore, we explored the molecular mechanism underlying the protective function of hUC‐MSC‐derived exosomes against LPS‐induced neuroinflammation.

## 2 METHODS

### 2.1 Materials

Dulbecco's modified Eagle's medium (DMEM), phosphate‐buffered saline (PBS), penicillin–streptomycin, fetal bovine serum (FBS), MitoSOX™ Red, MitoTracker™ Green FM, and various fluorescently conjugated secondary antibodies were purchased from Thermo Fisher (Thermo Fisher Scientific). Extracellular vesicle‐depleted FBS medium was purchased from SBI (System Biosciences). LPS (*Escherichia coli* 0111: B4) and H_2_O_2_ were purchased from Sigma (St. Louis). A protein extraction kit, bicinchoninic acid (BCA) protein assay kit, DAPI, and actin‐tracker green kit were purchased from the Beyotime Institute of Biotechnology (Beyotime, Haimeng, China). Primary antibodies against Interleukin‐6 (IL‐6, 21,865‐1‐AP), Tumor necrosis factor‐α (TNF‐α, 17,590‐1‐AP), NLRP3 (10904‐1‐AP), IBA1 (10904‐1‐AP), ARG1 (16001‐1‐AP), COX2 (12375‐1‐AP), NRF2 (16396‐1‐AP), Caspase‐1 (22915‐1‐AP), Lamin B1 (12987‐1‐AP), and GAPDH (60004‐1‐Ig) were obtained from Proteintech. Primary antibodies against iNOS (sc‐7271), Flotillin‐1 (sc‐74,566), CD63 (sc‐5275), GM130 (sc‐55,590), Calnexin (sc‐23,954), and TSG101 (sc‐7964) were purchased from Santa Cruz Biotechnology. Antibodies for NF‐κB p65 (8242), phospho‐NF‐κB p65 (3033), and ASC (67824S) were purchased from Cell Signaling Technology. The antibody for CD86 (553689) was obtained from BD Biosciences. The antibody against CD206 (AF2535) was purchased from R&D Systems, and the primary antibody against YM1 (ab192029) was purchased from Abcam. Proteintech (Wuhan, China) provided secondary antibodies for western blotting. A PKH26 Red Fluorescent Cell Linker Kit was obtained from MaoKang Biotechnology (Shanghai, China). The NF‐κB pathway inhibitor BAY 11–7082, NRF2 inhibitor ML385, and ROS scavenger N‐acetylcysteine (NAC) were purchased from Medchem Express. All other chemicals used in this study were of analytical grade.

### 2.2 Preparation and characterization of exosomes

hUC‐MSCs were purchased from Cyagen Biotechnology Inc. The culture medium used to expand cells was DMEM/F12 (Gibco, USA) containing 10% FBS (Gibco), and culturing was performed at 21% O_2_, 5% CO_2_, and 37°C. Cells at passage 5 were used for the experiment. When hUC‐MSCs reached 80% confluency, they were incubated with 0.25% trypsin–EDTA solution for 2 min. After adding a 2 mL culture medium to stop the trypsin digestion, the cells were centrifuged at 1000 rpm for 3 min. Subsequently, they were cultured in exosome‐depleted medium at 1 × 10^5^/mL, and 24 h later, the medium was collected. They were isolated from the medium using multiple ultracentrifugation steps, and the exosome pellet was suspended in 1000 μL lysis buffer or sterile PBS, depending on subsequent experiments. The concentration and size distribution of the isolated exosomes were confirmed via the nanoparticle tracking analysis using the NanoSight NS300 (Malvern Panalytical). The morphology was detected using transmission electron microscopy (TEM). Western blotting was performed to detect the levels of exosome markers, such as Flotillin‐1, CD63, and TSG101.

### 2.3 Microglia cell cultures and treatment

#### 2.3.1 BV‐2 cell lines

BV‐2 microglia cells were obtained from the Cell Bank of the Chinese Academy of Sciences (Shanghai, China). The cells were cultured in F12/DMEM with 10% FBS and 1% penicillin–streptomycin following the recommendations of the American Type Culture Collection. BV‐2 cells at 80% confluency were pretreated with hUC‐MSC‐derived exosomes (10 μg/mL) for 2 h and then with 500 ng/mL LPS or 150 μM H_2_O_2_ ^21^ for 12 h.

The BV‐2 cells were pretreated with NAC 5 mM for 1 h, followed by exposure to LPS (500 ng/mL) for 12 h.^22^ Based on a previous study,^23^ the BV‐2 cells were also pretreated with 5 nM of ML385, a specific inhibitor of NRF2, for 2 h before exosome treatment.

#### 2.3.2 Primary microglia culture

Neonatal mice (3 days after birth), purchased from Shanghai JieSiJie Laboratory Animals Ltd, were used to isolate primary microglia. Briefly, each neonatal mouse was rinsed in 75% ethanol and cold PBS to ensure sterile conditions, and the brain was isolated using a surgical scissors. The meninges and blood vessels were carefully stripped away under a light microscope (Leica DM 2500, Leica Microsystems GmbH, Germany). Subsequently, the brain tissues were minced with scissors and digested with 0.25% trypsin for 20 min at 37°C. Digestion was terminated by adding equal volume of culture medium. The mixed cell suspension was filtered using a 40‐μm cell strainer and centrifuged at 1000 rpm for 5 min. A single‐cell suspension of the mixed cells was seeded into 75‐cm^2^ flasks coated with 0.05 mg/mL poly‐L‐lysine (Sigma Aldrich, P9155). The mixed cell culture supernatant was removed the next day. On day 7, the culture flask was placed on a shaker at 180 rpm for 2 h at 37°C, and subsequently, the supernatant was centrifuged at 1000 rpm for 5 min. The cells were then used in subsequent experiments. The treatment for the primary microglial cultures was the same as that for BV‐2 cells.

### 2.4 hUC‐MSC‐derived exosome uptake in vitro

The red fluorescent dye PKH26 (MaoKang Biotechnology, Shanghai, China) was used to label exosomes according to the manufacturer's instructions. The labeled exosomes were co‐cultured with BV‐2 cells or primary microglial cultures at a concentration of 10 μg/mL for 6 h, 12 h, and 24 h. Subsequently, the cells were fixed with 4% paraformaldehyde for 40 min and blocked using 5% bovine serum albumin for 1 h at room temperature. The cytoskeleton was stained with Actin‐Tracker Green and the nucleus with DAPI. A confocal laser‐scanning microscope (Leica TCS SP 5, Leica Microsystems GmbH) was used for imaging.

### 2.5 In vivo exosome injection and brain tissue preparation

All animal experiments were approved by the Institutional Animal Care and Use Committee of Fudan University (number 202201011S). Adult male C57BL/6J mice (SPF grade) were obtained from Shanghai JieSiJie Laboratory Animals Ltd and housed in standard cages at 20–25°C, 40 ± 5% humidity, and light–dark cycle of 12 h:12 h.

PKH26‐labeled exosomes were injected into the mouse tail vein to validate the uptake of hUC‐MSC‐derived exosomes in vivo. The mice in the sham group were injected with saline only. For subsequent experiments, based on previous studies (30), 18 adult male mice (8 weeks old) were randomly divided into three groups: (1) the control group (*n* = 6) was intravenously administered 100 μL saline for 7 days; (2) the LPS group (*n* = 6) was intraperitoneally injected with 0.5 mg/kg LPS once a day for 7 days; and (3) the Exos+LPS group (*n* = 6) was injected with exosomes (40 μg/100 μL)^24^, ^25^ 1 h prior to LPS treatment. The mice in all groups were perfused with ice‐cold PBS at the end of the experiment. Subsequently, the hippocampal tissues were harvested for western blotting, or brain tissues were fixed using 4% paraformaldehyde for immunofluorescence and histology analyses.

### 2.6 ROS assay

Intracellular ROS were detected via 2,7‐dichlorodi‐hydrofluorescein (DCFH) staining. After the BV‐2 cells were treated with H_2_O_2_/LPS and/or exosomes for 12 h, the cells were washed three times with PBS and incubated in a medium containing 2.5 M DCFH at 37°C for 30 min. Subsequently, they were washed three times with PBS and stained with DAPI for 15 min.

For mitochondrial ROS measurements, the cells were washed three times with PBS after LPS treatment and incubated with DMEM medium containing 5 μM MitoSox red in the dark for 30 min at 37°C. Subsequently, the cells were incubated with DMEM medium containing 200 nM MitoTracker green in the dark for 30 min at 37°C.

ROS images were captured using a confocal microscope and analyzed using Image Pro Plus 6.0 (Media Cybernetics Co.A).

### 2.7 Immunofluorescence analysis

BV‐2 cells or primary microglia cultures were washed three times with PBS and fixed with 4% paraformaldehyde for 20 min at room temperature. Subsequently, 0.1% TritonX‐100 was used to permeabilize the cells for 10 min, followed by blocking in 5% BSA for 40 min and incubation with primary antibodies at 4°C overnight. The next day, the cells were washed three times with PBS and incubated with secondary antibodies for 2 h at room temperature. After washing three times with PBS, the cells were incubated with DAPI for 10 min to stain the nucleus. Images were captured using a confocal fluorescence microscope and analyzed via Image Pro Plus 6.0 (Media Cybernetics Co.).

The hippocampal slices were rinsed with PBS containing 0.1% Triton X‐100 for 40 min and blocked with 5% BSA for 60 min at room temperature. Subsequently, the sections were incubated with primary antibodies overnight at 4°C. After washing three times with PBS, the sections were incubated with the corresponding secondary antibodies for 1 h at room temperature. A fluorescence microscope with a Nikon DS‐U3 imaging system (Nikon Corp.) was used to capture images of the brain sections.

### 2.8 Western blot

The total protein content of the cells or hippocampal tissues was isolated using radioimmunoprecipitation assay (RIPA) lysis buffer. The protein concentration was determined using a BCA kit. The proteins in the nucleus and cytoplasm were extracted using a Nuclear and Cytoplasmic Protein Extraction Kit (Beyotime, Shanghai, China) according to the manufacturer's instructions. Equal amounts of protein were separated via sodium dodecyl sulfate‐polyacrylamide gel electrophoresis and transferred to a polyvinylidene fluoride membrane. The membrane was blocked using 5% skim milk for 1 h at room temperature and incubated with primary antibodies at 4°C overnight. The next day, it was washed three times with Tris‐buffered saline with 0.1% Tween® 20 and incubated with secondary antibodies for 1 h at room temperature. All proteins were visualized using enhanced chemiluminescence reagent, and relative protein levels were normalized to those of glyceraldehyde 3‐phosphate dehydrogenase (GAPDH) using the ImageJ software (NIH Image J system).

### 2.9 Statistical analysis

The data are expressed as the mean ± SD. Shapiro–Wilk test was used to analyze the normal distribution. The differences between groups were analyzed using one‐way analysis of variance, and differences were considered significant at *p* < 0.05. All statistical analyses were performed using GraphPad Prism 6.0 (GraphPad Software Inc.).

## 3 RESULTS

### 3.1 Characterization of hUC‐MSC‐derived exosomes

**FIGURE 1:**
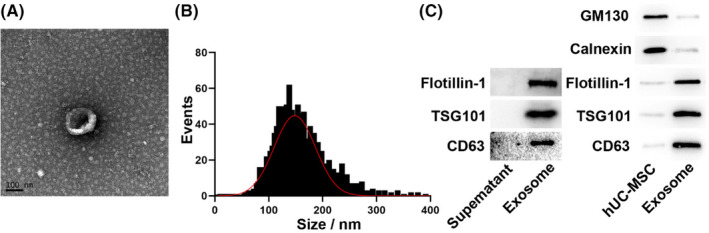
Isolation and characterization of exosomes derived from human umbilical cord mesenchymal stem cells. (A) Exosome morphology was captured using transmission electron microscopy. (B) Exosome size was quantified via nanoparticle tracking analysis. (C) Western blot was used to detect exosome‐positive (Flotillin‐1, TSG101, and CD63) and exosome‐negative (Calnexin and GM‐130) markers.

TEM revealed that the exosomes exhibited a typical elliptical shape (Figure 1A), and the nanosystem indicated that they had a mean diameter of 148.3 nm (Figure 1B). The initial concentration of exosomes was 3.2 × 10^10^ particles/mL. Western blot analysis indicated that these vesicles contained exosomal markers, including CD63, TSG101, and Flotillin‐1 (Figure 1C). The Calnexin and GM‐130 levels were negligible in the purified exosomes, suggesting that these preparations contained no endoplasmic reticulum or Golgi contamination (Figure 1C). Collectively, our results confirmed the successful isolation of hUC‐MSC‐derived exosomes.

### 3.2 Exosomes alleviate LPS‐ and H_2_O_2_‐induced oxidative stress and inflammation in BV‐2 Cells

**FIGURE 2:**
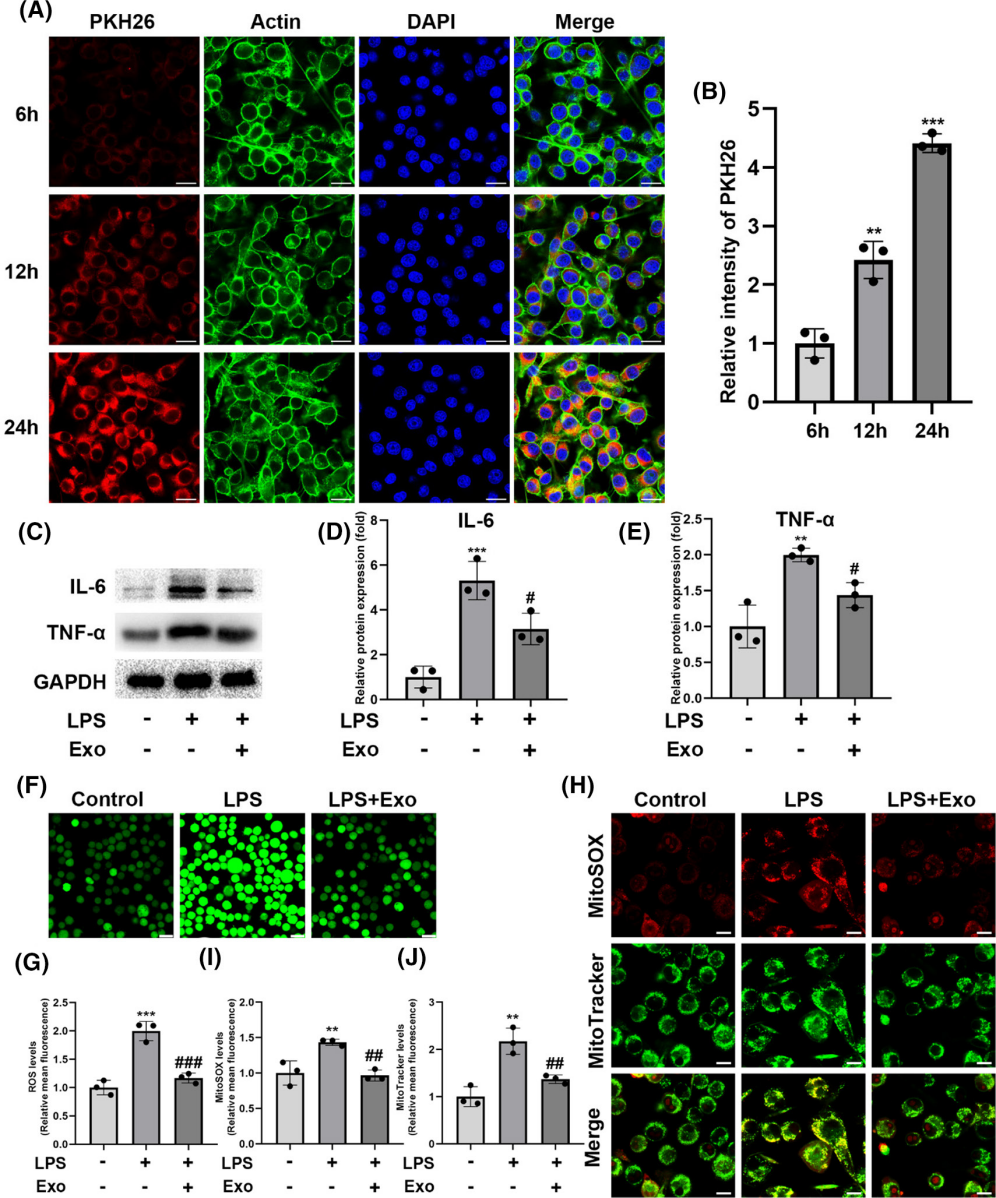
Anti‐inflammatory and antioxidant effects of human umbilical cord mesenchymal stem cell (hUC‐MSC)‐derived exosomes on lipopolysaccharide (LPS)‐induced neuroinflammation in vitro. (A and B) Immunofluorescence staining of BV‐2 cells and hUC‐MSC‐derived exosomes (PKH26) showed that the exosomes gathered inside the BV‐2 cells; scale bar = 25 μm. (C–E) Western blot analysis of IL‐6 and TNF‐α in BV‐2 cells after hUC‐MSC‐derived exosome treatment. (F and G) Intracellular reactive oxygen species (ROS) were labeled with the DCFH‐DA probe and examined via confocal microscopy; scale bar = 25 μm. (H–J) Mitochondrial ROS levels in BV‐2 cells treated with LPS. The mitochondrial mass and mitochondrial ROS were detected via Mitotracker Green/MitoSOX Red staining; scale bar = 10 μm; *n* = 3; mean ± SD; *p < 0.05; **p < 0.01; ***p < 0.001, compared with the control group; #*p* < 0.05; ##*p* < 0.01; ###*p* < 0.001, compared with the LPS group.

Confocal microscopy revealed that exosomes gradually gathered in the BV‐2 cells after co‐culturing the labeled exosomes with BV‐2 cells for 6 h, 12 h, and 24 h (Figure 2A,B), suggesting that they were gradually taken up by BV‐2 cells. Furthermore, western blot analysis indicated that LPS stimulation increased the levels of the pro‐inflammatory cytokines TNF‐α and IL‐6 in BV‐2 cells. However, the exosome treatment counteracted the LPS‐induced increase in TNF‐α and IL‐6 levels compared with their levels in non‐treated cells (Figure 2C–E). Intracellular ROS measurements demonstrated that the exosome treatment also decreased the ROS levels compared with those in the LPS group (Figure 2F,G). LPS administration significantly decreased NRF2, an anti‐inflammatory and antioxidant factor, which was reversed upon exosome treatment.

Huang et al. reported that H_2_O_2_ increases the inflammatory cytokine levels.^26^ We found that H_2_O_2_ treatment significantly increased the cellular ROS levels, whereas pretreatment with exosomes reversed this effect. The NRF2 inhibitor ML385 decreased NRF2 levels and abrogated the anti‐oxidative and anti‐inflammatory effects of exosome treatment.

Mitotracker Green and MitoSOX Red double staining may be used to indicate mitochondrial function.^27^ In this study, the LPS treatment increased mitochondrial ROS and mitochondrial mass. In contrast, exosome treatment reversed this effect (Figure 2H–J).

### 3.3 Exosomes induce microglia polarization from the pro‐inflammatory to anti‐inflammatory phenotype

**FIGURE 3:**
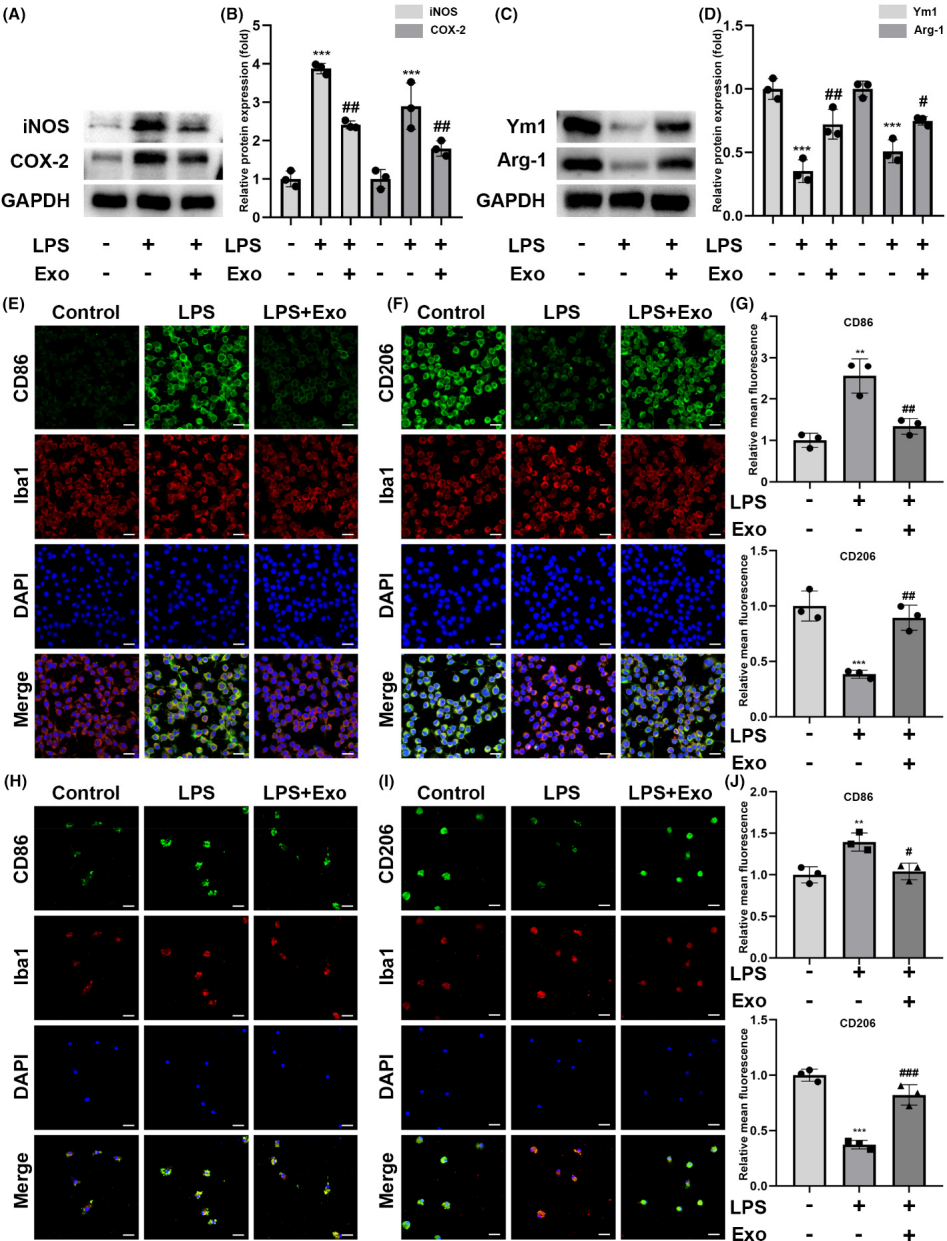
Effect of human umbilical cord mesenchymal stem cell‐derived exosomes on the microglia cell polarization. (A and B) Western blot analysis and quantification of iNOS and COX‐2. (C and D) Western blot analysis and quantification of YM1 and ARG1. (E) Representative images of microglia bearing a pro‐inflammatory (M1) phenotype in each group were examined using anti‐CD86 (green) and anti‐Iba‐1 (red) antibodies, as well as DAPI (blue); scale bar = 25 μm. (F) Representative images of anti‐inflammatory phenotype microglia in each group were examined using anti‐CD206 (green), and anti‐Iba‐1 (red) antibodies, as well as DAPI (blue); scale bar = 25 μm. (G) Relative fluorescence intensity analysis for CD86 and CD206. (H) Representative images of primary microglia bearing a pro‐inflammatory phenotype in each group were examined using anti‐CD86 (green), anti‐Iba‐1 (red) antibodies, and DAPI (blue); scale bar = 25 μm. (I) Representative images of anti‐inflammatory phenotype primary microglia in each group were examined by using anti‐CD206 (green) and anti‐Iba‐1 (red) antibodies, as well as DAPI (blue); scale bar = 25 μm. (J) Relative fluorescence intensity analysis for CD86 and CD206 in primary microglia; *n* = 3; mean ± SD; *p < 0.05; **p < 0.01; ***p < 0.001, compared with the control group; #*p* < 0.05; ##*p* < 0.01; ###*p* < 0.001, compared with the LPS group.

As shown in Figure 3A,B, the M1‐associated markers in LPS‐stimulated BV‐2 cells were upregulated, whereas the M2‐associated markers ARG1 and YM1 were downregulated compared with those in the control group. However, exosome treatment significantly decreased the pro‐inflammatory and increased the anti‐inflammatory marker levels compared with LPS treatment alone (Figure 3C,D). Additionally, exosome administration downregulated the pro‐inflammatory surface marker CD86 and upregulated the anti‐inflammatory surface marker CD206 in BV‐2 cells after LPS treatment (Figure 3E–G). For primary microglia, we sought to identify the purity of the primary cultured microglia. Similar to BV‐2 cells, exosome treatment regulated the abundance of CD86 and CD206 (Figure 3H–J) in primary microglia. These results indicated that LPS‐stimulated hUC‐MSC‐derived exosomes altered microglia polarization from a pro‐inflammatory (M1) to an anti‐inflammatory (M2) phenotype.

We further investigated the effects of the ROS scavenger NAC on LPS‐induced activation of BV‐2 cells. We found that NAC inhibited the LPS‐induced microglia polarization, suggesting that hUC‐MSC‐derived exosomes switch the microglia phenotype from pro‐inflammatory (classical M1) to anti‐inflammatory (classical M2) by inhibiting ROS production.

### 3.4 Exosomes decrease NLRP3 inflammasome activation by inhibiting the NF‐κB pathway in vitro

**FIGURE 4:**
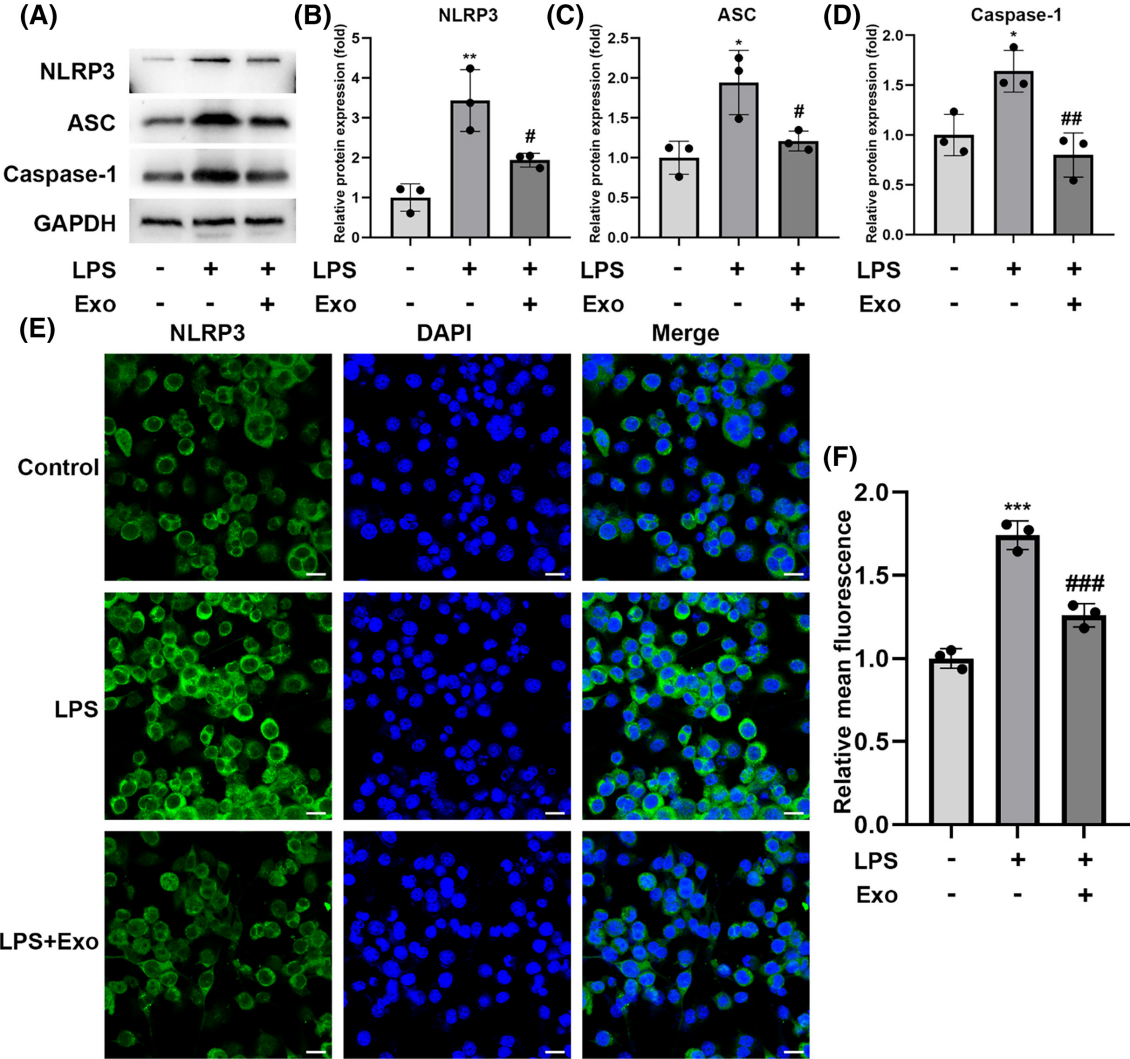
Human umbilical cord mesenchymal stem cell‐derived exosomes inhibit NLRP3 inflammasome activation in BV‐2 cells. (A–D) Protein levels of NLRP3, ASC, and Caspase‐1 after exosome treatment were detected using western blotting. (E and F) The NLRP3 inflammasome in BV‐2 cells was detected via immunofluorescence; scale bar = 25 μm; *n* = 3; mean ± SD; *p < 0.05; **p < 0.01; ***p < 0.001, compared with the control group; #*p* < 0.05; ##*p* < 0.01; ###*p* < 0.001, compared with the LPS group.

NLRP3, ASC, and Caspase‐1, increased after LPS exposure, however, exosome treatment significantly decreased the LPS‐induced upregulation of these proteins in BV‐2 cells (Figure 4A–D). hUC‐MSC‐derived exosomes reduced the NLRP3 levels compared with those of LPS treatment only (Figure 4E,F). These results demonstrated that exosomes suppressed the activation of NLRP3 inflammasomes to alleviate neuroinflammation.

**FIGURE 5:**
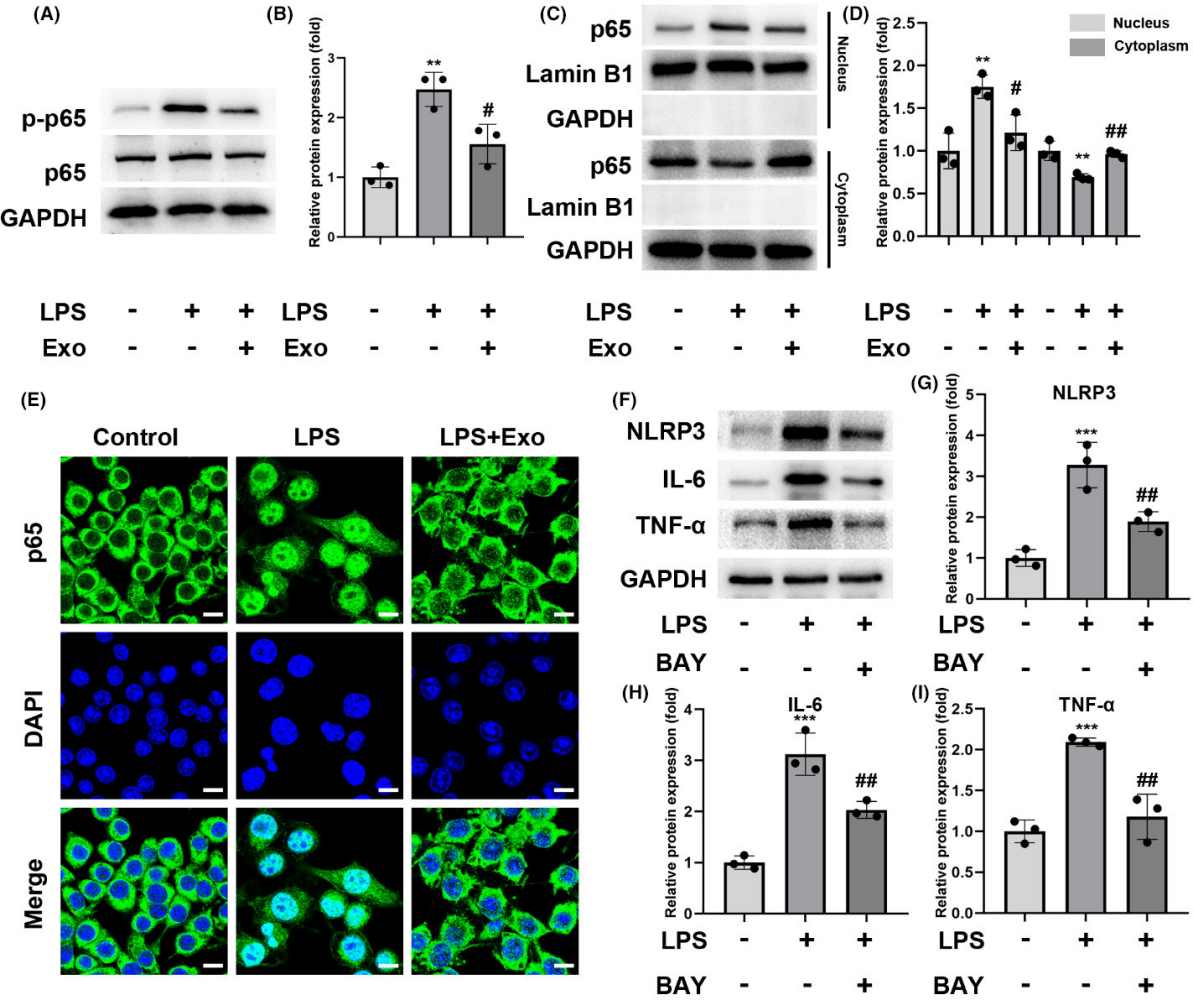
Contribution of NF‐κB P65 to the function of human umbilical cord mesenchymal stem cell‐derived exosomes in the BV‐2 cells. (A and B) The levels of p65 and those of its phosphorylated form (p‐p65) were detected via western blotting. (C and D) The p65 nuclear translocation was determined via western blotting. (E) Immunofluorescence demonstrating the nuclear location of p65 in BV‐2 cells; scale bar = 10 μm. (F–I) The levels of NLRP3, IL‐6, and TNF‐α in different groups after administration of the NF‐κB pathway inhibitor BAY 11–7082 were detected via western blot; *n* = 3; mean ± SD; *p < 0.05; **p < 0.01; ***p < 0.001, compared with the control group; #*p* < 0.05; ##*p* < 0.01; ###*p* < 0.001, compared with the LPS group.

The NF‐κB signaling pathway regulates inflammatory responses, therefore, we monitored the expression of NF‐κB p65 and its phosphorylation to an activated form.^28^ LPS stimulation increased the expression of phospho‐NF‐κB p65, whereas exosomes inhibited the expression of phospho‐NF‐κB p65 (Figure 5A,B). NAC treatment inhibited the LPS‐induced phosphorylation of NF‐κB p65, suggesting that oxidative stress might participate in p65 phosphorylation. The inhibition of ROS by NAC also decreased the TNF‐α and IL‐6 levels in LPS‐induced cells. Xue et al. found that the lincRNA‐Cox2 promoted NF‐κB p65 nuclear translocation and transcription, which modulated the expression of the inflammasome sensor *NLRP3* and adaptor *ASC*,^29^ thus, we performed nucleocytoplasmic separation to compare the NF‐κB p65 levels in the nucleus and cytoplasm and observed that LPS promoted the nuclear translocation of NF‐κB p65. In contrast, the exosomes significantly reduced the LPS‐induced nuclear translocation of NF‐κB p65 (Figure 5C–E).

Subsequently, we used the NF‐κB p65 inhibitor BAY 11–7082 (10 μM) to determine whether NF‐κB signaling mediates the activation of NLRP3 inflammasomes. NF‐κB p65 inhibition significantly reduced the LPS‐mediated upregulation of NLRP3 as well as cytokines IL‐6 and TNF‐α (Figure 5F–I). Further, we showed that the NRF2 inhibitor ML385 blocked the anti‐inflammatory effects of hUC‐MSC‐derived exosomes in LPS‐stimulated BV‐2 cells. Collectively, these findings suggest that exosomes inhibit the activation of NLRP3 via the Nrf2/NF‐κB signaling pathway.

### 3.5 Exosomes alleviate LPS‐induced neuroinflammation in mice brains

**FIGURE 6:**
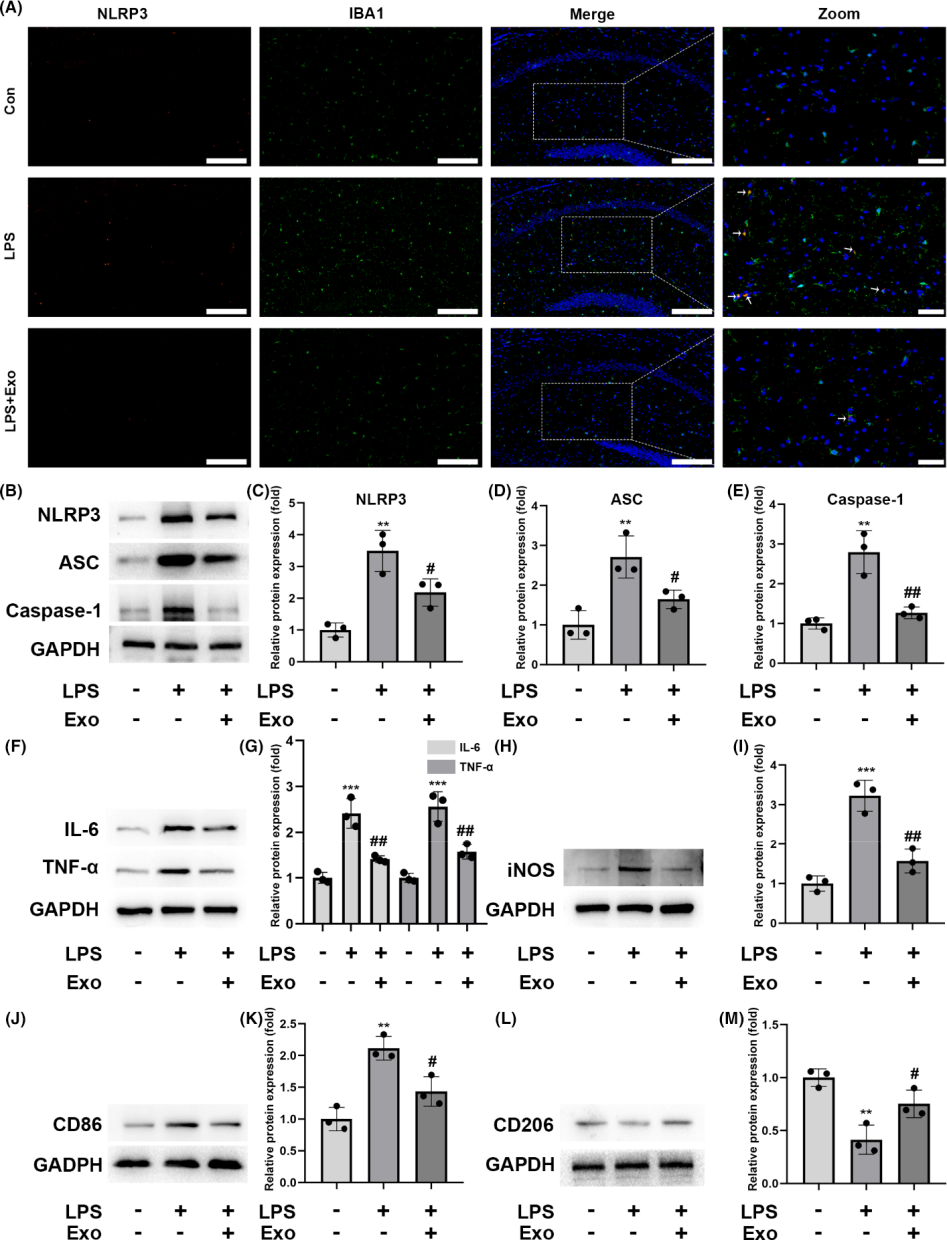
Effect of human umbilical cord mesenchymal stem cell‐derived exosomes on neuroinflammation in vivo. (A) Representative photomicrographs of NLRP3 and Iba‐1 positive cells in the hippocampus. White arrows indicate the colocalization (yellow) of NLRP3 (red) with IBA1 (green). Low‐magnification scale bar = 200 μm, high‐magnification scale bar =50 μm. (B–E) NLRP3, ASC, and Caspase‐1 levels in the hippocampus, measured via western blotting. (F and G) Western blot analysis of the protein levels of IL‐6 and TNF‐α in the hippocampus. (H and I) Protein levels of iNOS in the hippocampus were measured via western blotting. (J and K) The M1 marker CD86 was measured via western blotting. (L and M) The M2 marker CD206 was measured via western blotting; *n* = 6; mean ± SD; *p < 0.05; **p < 0.01; ***p < 0.001, compared with the control group; #*p* < 0.05; ##*p* < 0.01; ###*p* < 0.001, compared with the LPS group.

To evaluate the effects of hUC‐MSC‐derived exosomes on neuroinflammation in vivo, the exosomes were labeled with the red fluorescent dye PKH26 and injected through the tail vein. After 24 h, we successfully detected exosomes in the cortex and hippocampus; however, we could not detect PKH26‐labeled exosomes in the sham group. As a marker of activated microglia, IBA1 levels were significantly increased in the hippocampus upon LPS treatment compared with those in the control group. In contrast, exosome treatment significantly abolished the LPS‐induced increase in IBA1 abundance in the hippocampus (Figure 6A).

Furthermore, colocalization studies showed that LPS triggered the upregulation of NLRP3 in the hippocampal microglia, whereas the administration of exosomes counteracted this effect (Figure 6A). LPS stimulation increased the levels of NLRP3, ASC, and Caspase‐1 in the hippocampus, whereas exosomes significantly prevented their increase (Figure 6B–E). The IL‐6 and TNF‐α levels were upregulated upon LPS treatment in vivo but decreased upon hUC‐MSC‐derived exosome administration (Figure 6F,G). Furthermore, the administration of exosomes significantly reduced the LPS‐mediated increase in iNOS levels (Figure 6H,I). Similar to cells, exosomes promoted hippocampal microglia anti‐inflammatory (M2) polarization (Figure 6J–M), and the CD86 and CD206 stainings were also similar.

## 4 CONCLUSION

As potential cell therapy agents, hUC‐MSCs confer therapeutic benefits by secreting various small soluble molecules, including exosomes.^30^, ^31^ In the present study, we demonstrated that hUC‐MSC‐derived exosomes could alleviate LPS‐ or H_2_O_2_‐induced neuroinflammation and oxidative stress in vitro and in vivo, evidenced by the inhibition of NLRP3 inflammasome activation and downregulation of pro‐inflammatory cytokine levels. Mechanistically, exosomes inhibit the NRF2/NF‐κB p65/NLRP3 inflammasome signaling pathway to alleviate neuroinflammation and oxidative stress, as well as promote a shift from a pro‐inflammatory microglia phenotype (M1) to an anti‐inflammatory one (M2). Therefore, our study provides experimental evidence that hUC‐MSC‐derived exosomes possess therapeutic potential for neuroinflammation‐associated diseases.

Exosomes are small bioactive vesicles secreted from various cell types that transmit signals between cells. Exosomes from different sources have been used to treat several diseases,^32^ and the therapeutic effects of hUC‐MSC‐derived exosomes have been evaluated for several kidney diseases, Alzheimer's disease, inflammatory bowel disease, spinal cord injury, and acute myocardial ischemia.^33^ Exosome extraction by ultracentrifugation reduces the cost and risk of contamination and allows for the simultaneous processing of large sample volumes.^34^ We successfully obtained and characterized sufficient exosomes to perform in vitro and in vivo studies. The isolation of hUC‐MSC exosomes by ultracentrifugation is highly efficient and convenient, therefore, this exosome therapy strategy has considerable clinical translational value.^35^

Few studies have investigated the use of MSC‐derived exosomes for neuroinflammation control. Zhang et al. showed that hUC‐MSC‐derived exosomes could reduce microglial‐mediated neuroinflammation and, thus, infarct volume and behavioral deficits in an ischemic stroke animal model.^36^ Dong et al. demonstrated that bone marrow MSC‐secreted exosomes attenuated the microglia activation, induced by middle cerebral artery occlusion, and M1 polarization, as well as upregulated the expression of pro‐inflammatory factors.^37^ These effects are regulated by microRNAs, carried by exosomes. Our results that hUC‐MSC‐derived exosomes can downregulate LPS‐induced inflammation in the microglia and mouse brain by means of oxidative stress inhibition are in accordance with another study, in which exosomes secreted by bone marrow MSCs modulated the LPS‐stimulated microglial M1/M2 polarization and alleviated the inflammation‐mediated neurotoxicity.^38^

LPS are endotoxins that commonly induce SAE and NF‐κB expression by binding to toll‐like receptor 4 (TLR4).^39^ LPS cause neurotoxicity to microglia, which acquires a pro‐inflammatory (M1) phenotype.^40^ Activated microglia produces higher levels of pro‐inflammatory cytokines, which exacerbate neuronal damage. In contrast, the alternative anti‐inflammatory (M2) phenotype is characterized by the release of protective cytokines, which alleviates neuroinflammation and promotes tissue restoration.^41^ Therefore, the classical M1/M2 polarization is widely used for assessing whether neuroinflammation is relieved in in vitro and in vivo models.^42^ Using multiple approaches, we demonstrated that hUC‐MSC‐derived exosomes promoted a shift from a pro‐inflammatory to an anti‐inflammatory phenotype in LPS‐stimulated microglia.

Bone marrow MSC‐derived exosomes regulate the acquisition of microglia phenotypes,^43^ promote remyelination, and reduce neuroinflammation.^44^ Exosomes may help maintain brain homeostasis, and therefore, they bear promising therapeutic potential for neurodegenerative diseases.^45^

The beneficial effects of exosomes involve the IDO/Treg and FasL/Fas,^46^ AK1/TRAF6,^36^ and the NLRP3‐related signaling pathways.^47^ The NLRP3 inflammasome, a critical regulator of inflammation and infection, has been studied widely.^48^ We observed that LPS increased NLRP3 and IL‐1β protein levels while exosome treatment downregulated the levels of inflammasome‐related proteins. The NF‐κB signaling pathway is involved in NLRP3 activation. LPS activate NLRP3 in BV2 cells by translocating NF‐κB p65 from the cytoplasm to the nucleus.^49^ There, it regulates the expression of several inflammatory mediators, which cause neuronal receptors to activate protein kinases, trigger inflammation, and accelerate neurodegeneration.^50^

We explored the mechanism underlying the anti‐neuroinflammatory and anti‐oxidative effects of hUC‐MSC exosomes. Increased ROS generation activated the microglia, whereas reduced mitochondrial ROS accumulation alleviated the NLRP3 inflammasome activation.^51^ ROS scavengers also inhibited NF‐κB p65phosphorylation, whereas an NF‐κB p65 inhibitor decreased NLRP3 activation. Therefore, inhibition of ROS production may participate in exosome‐mediated inhibition of NLRP3 activation and regulation of microglia polarization.

The transcription factor NRF2 has been reported to interact with NF‐κB p65 to exert anti‐inflammatory and anti‐oxidative effects.^52^ Our findings indicated that exosome treatment attenuated oxidative stress and inflammation by increasing the NRF2 levels. This evidence indicates that the NRF2/NF‐κB/NLRP3 pathway mediates the anti‐oxidative and anti‐neuroinflammatory effects of hUC‐MSC‐derived exosomes. Further studies should investigate whether hUC‐MSC‐derived exosome treatment could improve neurobehavioral outcomes in neuroinflammation‐associated brain diseases.

## CONFLICT OF INTEREST STATEMENT

The authors declare that they have no conflicts of interest in association with the present study.

## Data Availability

The data that support the findings of this study are available from the corresponding author upon reasonable request.
